# Fibroblasts and Their Pathological Functions in the Fibrosis of Aortic Valve Sclerosis and Atherosclerosis

**DOI:** 10.3390/biom9090472

**Published:** 2019-09-10

**Authors:** Savita Singh, Michael Torzewski

**Affiliations:** 1 Dr. Margarete Fischer-Bosch-Institute of Clinical Pharmacology and University of Tuebingen, 70376 Stuttgart, Germany; savita.singh@ikp-stuttgart.de; 2 Department of Laboratory Medicine and Hospital Hygiene, Robert-Bosch-Hospital, 70376 Stuttgart, Germany

**Keywords:** atherosclerosis, aortic valve stenosis, fibrosis, fibroblasts

## Abstract

Cardiovascular diseases, such as atherosclerosis and aortic valve sclerosis (AVS) are driven by inflammation induced by a variety of stimuli, including low-density lipoproteins (LDL), reactive oxygen species (ROS), infections, mechanical stress, and chemical insults. Fibrosis is the process of compensating for tissue injury caused by chronic inflammation. Fibrosis is initially beneficial and maintains extracellular homeostasis. However, in the case of AVS and atherosclerosis, persistently active resident fibroblasts, myofibroblasts, and smooth muscle cells (SMCs) perpetually remodel the extracellular matrix under the control of autocrine and paracrine signaling from the immune cells. Myofibroblasts also produce pro-fibrotic factors, such as transforming growth factor-β1 (TGF-β1), angiotensin II (Ang II), and interleukin-1 (IL-1), which allow them to assist in the activation and migration of resident immune cells. Post wound repair, these cells undergo apoptosis or become senescent; however, in the presence of unresolved inflammation and persistence signaling for myofibroblast activation, the tissue homeostasis is disturbed, leading to excessive extracellular matrix (ECM) secretion, disorganized ECM, and thickening of the affected tissue. Accumulating evidence suggests that diverse mechanisms drive fibrosis in cardiovascular pathologies, and it is crucial to understand the impact and contribution of the various mechanisms for the control of fibrosis before the onset of a severe pathological consequence.

## 1. Introduction

Atherosclerosis and aortic valve sclerosis (AVS) combined are the major cardiovascular diseases, comprising the highest mortality related to cardiovascular disease. Both the pathologies have similar risk factors, including hypertension, higher lipid levels, and smoking. Atherosclerosis has long been identified as an inflammatory disease with low-density lipoproteins (LDL) overload being the major trigger factor, and very recently, AVS has also been investigated as an inflammatory disease, contrary to the past view of it being a degenerative disease. Lesions of atherosclerosis occur primarily in large- or medium-sized elastic arteries; these lesions begin as fatty streaks and may be present throughout a person’s lifetime [1], and these atherosclerotic lesions consist of immune cells and smooth muscle cells (SMCs) as cellular components, together with the fatty streaks [2,3]. The fatty streaks in atherosclerosis can evolve to pathological fibrous plaques with the help of proliferating fibrous tissues, which produces increased amounts of connective tissue, leading to luminal narrowing. Fibrous plaques obstruct blood flow and rupturing of unstable plaques lead to clinical events such as strokes and thrombosis. Fibrosis is involved in both atherosclerosis and AVS, and fibroblasts are the major cell population involved in remodeling of the extracellular matrix. Fibroblasts originate from the differentiation of other cell populations, including stem cells and quiescent residential cells of the tissue in the fibrous plaques in atherosclerosis. Key functions of fibroblasts in atherosclerosis include regulation of inflammation [4,5], extracellular matrix (ECM), collagen production, and maintenance of the structural integrity of the plaque. In the initial phase of fibrosis, arteries with injured endothelium are remodeled, and the initial plaque formation resembles a protective process; however, as in all chronic inflammatory conditions, fibrotic components in the plaque produce excess cytokines and proteolytic enzymes, causing constrictive remodeling. Myofibroblasts and smooth muscle cells from media, and adventitial fibroblasts are the two major components for plaque remodeling. α-smooth muscle actin (α-SMA)-positive myofibroblasts-like phenotype has been identified in all stages of coronary lesions, including mildly stenotic lesions, stable stenotic plaques, and restenotic lesions [6,7]. Myofibroblasts in the plaque are likely derived from multiple sources, including adventitial fibroblasts [8], the endothelial to mesenchymal transition [9], and SMC to myofibroblast transition [10]. The process of differentiation varies depending on the disease pathology, microenvironment, and progressed stage of the disease.

**Figure 1:**
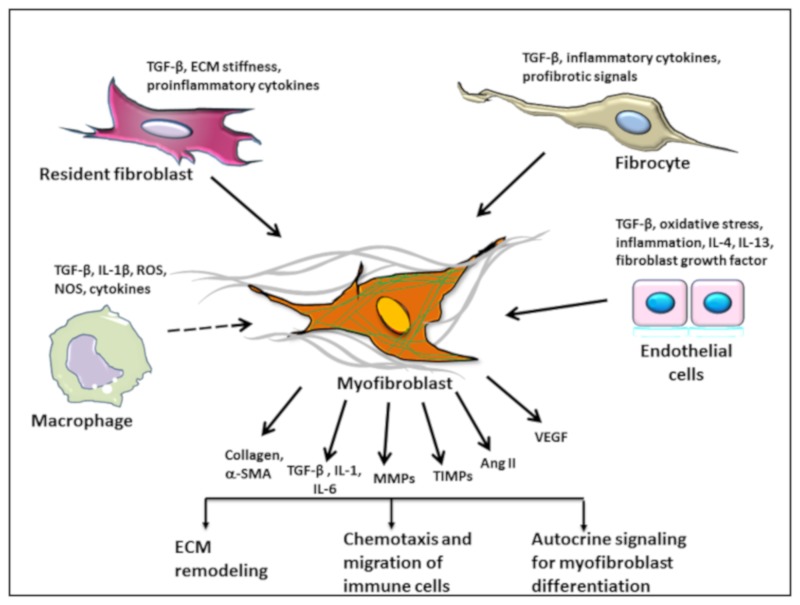
Various progenitor cells lead to the origin of myofibroblasts in atherosclerosis and aortic valve sclerosis. Resident fibroblasts in the tissue and fibrocytes undergo differentiation under the influence of TGF-β, ECM stiffness, proinflammatory cytokines, and profibrotic signals. Endothelial cells undergo an endothelial to mesenchymal transition (EndMT) under the influence of oxidative stress, inflammation, TGF-β, IL-4, IL-13, and fibroblast growth factor. TGF-β, IL-1β, ROS, NOS, and cytokines facilitate the activation of macrophages, which are a major source of profibrotic cytokines, leading to an indirect myofibroblast differentiation through hematopoietic cells. The relative contribution of various cell types to the myofibroblast population is variable and depends on the degree of fibrosis. TGF-β: transforming growth factor-β; ECM: extracellular matrix; IL-4: interleukin-4; IL-13: interleukin-13; α-SMA: α-smooth muscle actin; IL-1: interleukin-1; IL-6: interleukin-6; MMPs: matrix metalloproteases; TIMPs: tissue inhibitors of MMPs; Ang II: angiotensin II; VEGF: vascular endothelial growth factor; IL-1β: interleukin-1β; ROS: reactive oxygen species; NOS: nitric oxide synthase.

Similar pathological features occur in AVS, where damage to the endothelium causes activation and migration of fibroblasts to produce an ECM and facilitate matrix reorganization in response to locally secreted cytokines from resident cells of valve layer and immune cells. Persistent activation of these cells leads to valve thickening known as sclerosis, which occurs simultaneously with the infiltration of immune cells, deposition of lipids, proteoglycans, and eventual calcification, causing the stenosis of the valve. Damage to the endothelium is not only an initiating step but a major driving force in AVS. Endothelial injury triggers lipid infiltration and inflammatory responses, including infiltration of monocytes, macrophages, T cells, and mast cells. These immune cells secrete a plethora of cytokines, which facilitates the activation of fibroblasts and differentiation of cells of mesenchymal and endothelial origin into fibroblasts. Myofibroblasts have a very heterogeneous origin, which is explained in further details in Figure 1. However, the differentiation of myofibroblasts follows a specific sequence of events. Under normal conditions, fibroblasts exhibit a sparse ECM and minimal cell-cell or cell-matrix connections, which is altered under the impact of environmental cues such as matrix rigidity, transforming growth factor-β1 (TGF-β1) expression, and a specialized ECM. These stimuli trigger the differentiation of fibroblasts into α-SMA-positive myofibroblasts [11,12]. Enormous matrix reorganization is as important of a cause for fibrosis as the inflammatory cytokines. In the absence of remodeling and injury, fibroblasts are shielded by a cross-linked ECM, and this protection is lost under the conditions of matrix remodeling, which causes the fibroblasts to acquire actin-based contractile stress fibers and eventually complete the differentiation into myofibroblast phenotype with α-SMA expression. Activated fibroblasts produce matrix metalloproteases (MMPs), inhibitors of MMPs, TGF-β1, interleukin-1 (IL-1), interleukin-6 (IL-6), angiotensin II (Ang II), vascular endothelial growth factor (VEGF), collagen for an extracellular matrix, and fibronectin and laminin for the structural framework. These factors allow for the recruitment of immune cells, and the concerted action of proinflammatory cytokines from immune cells and myofibroblasts continue a perpetual myofibroblast differentiation, proliferation, and matrix remodeling. Moreover, increased proliferation of fibroblasts facilitates the formation of spacious and non-solid ground substance in the ECM, which allows for the recruitment of non-resident immune cells. Although there are numerous mechanisms involved in fibrosis, myofibroblasts appear to be one of the primary pathogenic cells [13] and therefore have been extensively studied for their tumorigenic phenotype in numerous cancers as well.

Despite major differences in the pathophysiology of atherosclerosis and AVS, fibrosis plays a central role, and in this review, we will summarize the molecular mechanisms involved in fibrosis in both the entities, including the recent understanding of the cell types involved and various pathways in myofibroblast differentiation.

## 2. Role of Fibroblasts in Atherosclerosis

Characterizing the resident cell population in blood vessels and valves is important to understand the cellular contribution and pathogenesis of the disease. The vascular wall in arteries consists of a three-layered structure: the innermost layer composed of endothelial cells (ECs) with dispersed smooth muscle cells is called intima, the middle layer is the tunica media, which consists of SMCs embedded in a collagen and proteoglycan matrix, while the outer layer, the adventitia is composed of fibroblasts with collagen and elastin arranged in a longitudinal network. Atherosclerosis is characterized by the accumulation of lipids and a collagen-rich extracellular matrix in the vessel wall, and advanced atherosclerotic plaques are characterized by a fibrous cap rich in SMCs and fibroblasts. These cells secrete proinflammatory cytokines and an ECM to facilitate the buildup of the plaque [1]. Moreover, fibroblasts in adventitia maintain plasticity for phenotypic conversion upon stimulation by cytokines and other external physiological stress, which allows them to remodel the vascular wall, e.g., TGF-β was shown to induce the differentiation of fibroblasts into myofibroblasts in a balloon overstretch coronary artery injury in a porcine model [14]. These myofibroblasts migrate to the neo-intima, where they contribute to collagen deposition and neointimal expansion [8]. This extensive remodeling determines the degree of stenosis and clinical manifestations of atherosclerosis.

## 3. Role of Fibroblasts in AVS

The aortic valve consists of a three-leaflet structure, except in the cases of congenital bicuspid aortic valves, where it consists of two leaflets. The leaflets are smaller than 1 mm in thickness and organized in three layers: fibrosa, spongiosa, and ventricularis. The fibrosa on the aortic side of the valve is the main load-bearing layer composed predominantly of collagen I. The spongiosa is composed of proteoglycans with scattered collagen fibers that assist in flexibility. The ventricularis is composed of elastin and collagen. Vascular endothelial cells (VECs) leaflet surfaces on both sides of the valve and the vascular interstitial cells (VICs) make up the major cellular component of the layers. Although VECs contribute to valve homeostasis and remodeling via regulating cellular permeability and the interstitial cell phenotype [15,16], it is the VICs that are the primary pathogenic cells in AVS. VICs are a heterogeneous population of fibroblasts with about 5% myofibroblasts and SMCs. Under normal conditions VICs are quiescent and maintain the structural integrity and function of the valve; however, under pathological conditions, the percentage of myofibroblasts increases to up to 30%, accompanied by the loss of structural integrity [17,18,19]. Myofibroblasts influence immune cell chemotaxis, migration, retention, and apoptosis, thereby acting as key effector cells in the persistence of inflammation. Different progenitor cells transform into myofibroblasts, which are discussed later in this article. Fibroblasts, once activated, cause a disorganized ECM and disruption of the tri-layer valve structure, which disrupts the proper mechanical function of the valve [20]. Although, most cases of AVS progress to calcific aortic valve disease, even mild fibrosis without calcification has been associated with improvised valve function and an increase in cardiovascular mortality [21].

## 4. Mechanisms of Fibrosis in AVS and Atherosclerosis

Aortic valve sclerosis can progress with varying degrees of fibrosis, calcification, and osteogenesis. Although all end-stage valves exhibit calcification, recent gender-based studies have identified distinct clinical phenotypes and post-surgical complications in men and women [22]. Females are reported to have a higher degree of fibrosis and lower calcification than males [23], suggesting the disease progresses via a multitude of mechanisms and modulators. Regardless of the fibrocalcific spectrum in AVS, fibrosis plays a major role, and therefore, it is essential to delineate different mechanisms. In this section, we will discuss the molecules involved in the fibrosis in AVS and how some of them are involved in atherosclerosis as well.

### 4.1. Transforming Growth Factor β

TGF-β1, -β2, and -β3 are members of the TGF-β superfamily of extracellular ligands, which are regulators of cellular proliferation, differentiation, and the extracellular matrix [24]. TGF-β isoforms are expressed by all the effector cells in AVS and atherosclerosis, including endothelial cells, macrophages, vascular smooth muscle cells, T cells, and fibroblasts [3]. As discussed in the introduction, TGF-β1 secreted by fibroblasts in valves has an autocrine and paracrine function. It regulates the differentiation of the myofibroblast phenotype through the induction of contractile forces and α-SMA expression [25], and collagen expression through the binding of smad3/smad4 to the TGF-β-responsive element (TbRE) within the *COL1A2* gene [26]. Although the Smad independent signaling activated by TGF-β that includes extracellular signal-regulated kinase 1/2 (ERK1/2), p38 mitogen-activated protein kinase (MAPK), c-Jun N-terminal kinase (JNK), and phosphatidylinositol 3-kinase (PI3K) has not been investigated in AVS so far, it is possible that TGF-β1 exerts its fibrotic function through a multitude of effector molecules. In atherosclerosis, TFG-β has been associated with both atherogenic and atheroprotective properties. Experimental studies related to atherogenic properties of TGF-β reported that TGF-β induced proteoglycan production in SMCs, as well as stabilized the atherosclerotic lesions. Additionally, inhibition of TFG-β signaling by anti-TGF-β antibodies reduced collagen content in the lesions, leading to exacerbation of atherosclerotic lesions [27]. Contrary to the reported atherogenic role, clinical data from as early as two decades ago has indicated a negative correlation between serum TGF-β1 levels and advanced atherosclerosis [28], and these findings have also been supported by recent experimental studies in mice models reporting an atheroprotective role of TGF-β [29,30]. TGF-β also regulates expression of α8 integrins in the neointima, which is required for contractile properties and communication with the extracellular matrix [31]. As TGF-β is a pleiotropic cytokine produced by multiple cell types, its impact on fibrosis is highly complex and context-dependent. Individual studies in any single-cell population are not sufficient to delineate the complex function of TGF-β. Complete inhibition of TFG-β activity is possibly not a viable option for controlling fibrosis in AVS due to its pleiotropic effects. However, inhibitors of the TGF-β receptor, which may decrease TGF-β activity, could be potentially beneficial.

### 4.2. Renin-Angiotensin System

Synthesis of Ang II and activation of the renin-angiotensin system is a key feature in aortic valve stenosis. Ang II is generated from angiotensin I by the action of angiotensin-converting enzyme (ACE) [5,32]. AT1, a receptor for Ang II, is expressed only in fibroblasts of sclerotic lesions [5], where their activation by Ang II mediates fibroblast proliferation, ECM production, cholesterol accumulation in macrophages, and increased oxidative stress [5,33]. ACE has been shown to co-localize in the sclerotic lesion with LDL and Ang II, and in resident macrophages in the lesions, where it stimulates the generation of ROS and promotes the LDL-induced pathogenesis of AVS [34]. Ang II negatively regulates the expression of MMP1 and enhances collagen accumulation in cardiac fibroblasts [35]. Unlike fibroblasts in sclerotic lesions, SMCs consistently express AT1, and Ang II has been known to trigger their proliferation and migration to the outer layer of the plaque, contributing to a negative remodeling of the vessel walls [36,37]. The concerted action of ROS and Ang II stimulates the differentiation of adventitial fibroblasts into myofibroblasts through the p38 MAPK and JNK pathway [38]. Ang II is also known to increase TGF-β1 expression in cardiac hypertrophy [39], as TGF-β also regulates p38 MAPK signaling; therefore, it may be possible that Ang II and TGF-β function cooperatively, as shown in the study by Schultz et al., which demonstrated TGF-β1 as an important mediator for Ang II-induced cardiac hypertrophy [40]. Although contradictory reports exist regarding the efficacy of ACE inhibitors in aortic stenosis [41,42], it may be possible to target the angiotensin pathway, particularly AT1 inhibition, to control fibrosis, and to at least partially inhibit the undesirable effects of TGF-β overexpression in the fibrosis of AVS.

### 4.3. Immune Cells and Cytokines

Expression of the pro-inflammatory cytokines, TGF-β, tumor necrosis factor α (TNFα), IL-1β, and IL-6 is consistently observed in atherosclerosis and AVS. Under the influence of inflammatory cytokines, fibroblasts themselves produce multiple cytokines and are responsive to many more, which induce their differentiation into myofibroblasts, as well as regulate the ECM organization. In the heart valves, fibroblasts have been shown to secrete TGF-β1 and activate TGF-β-Smad signaling upon mechanical injury, with a concurrent increase in α-SMC expression, which is indicative of the myofibroblast phenotype [43]. TGF-β and other cytokines secreted by VICs further facilitate the recruitment of immune cells. Thus, histopathological examination of stenotic heart valves has demonstrated the infiltration of monocytes, T cells, and macrophages. Monocytes secrete IL-1β, a potent pro-inflammatory cytokine, which is implicated in multiple pathologies including aortic valve stenosis [44]; interestingly IL-1β and monocytes have not been found in the normal aortic valves [45]. IL-1β induces expression of pro-inflammatory cytokines, IL-6, and IL-8 through nuclear factor kappa-light-chain-enhancer of activated B cells (NF-κβ); moreover, myofibroblasts stimulated with IL-1β show an increased expression of MMP-1, as well as other proteins involved in fibrosis, indicating a fibrogenic function of IL-1β [46]. In experimental models of cardiac fibrosis, IL-1β promoted matrix degradation by enhancing the expression of MMPs, decreasing collagen synthesis, and negatively regulating the synthesis of inhibitors of MMPs [47]. Similar effects of IL-1 in upregulating MMPs expression have been reported in atherosclerotic plaques, leading to matrix degradation and plaque destabilization [48]. Besides monocytes, macrophages that infiltrate the fibrotic areas are also a source of MMP-1, -7, -9, and -12. MMPs produced by macrophages contribute to matrix homeostasis and tissue remodeling. M2 macrophages also directly promote collagen I expression by producing L-proline required for collagen synthesis by fibroblasts [49,50]. TNFα produced by macrophages, b cells, and fibroblasts also contributes to fibrosis. TNFα induces the expression of TGF-β in fibroblasts [51], it also induces macrophage inflammatory protein 1-α (MIP-1α) production in macrophages through the induction of intracellular adhesion molecule 1 (ICAM) expression in the fibroblasts [52], which has been implicated in pulmonary fibrosis [53]. Furthermore, TNF-α has also been reported to trigger calcification in VICs through NF-κβ [54].

## 5. Origin of Myofibroblasts

Myofibroblasts originate from a range of progenitor cells in cardiac pathologies, as well as in cancers. Well-examined progenitor cells for myofibroblast differentiation include resident fibroblasts, fibrocytes, mesenchymal cells, and epithelial/endothelial cells. A marked increase in the mediators of fibroblast trans-differentiation post-injury and abundance of proliferating fibroblasts in the fibrosis of the heart indicates the induction of the activation of resident fibroblasts as a source of fibroblasts [55]. In addition to the activated resident fibroblasts, circulating fibrocytes have also been documented to mature into fibroblasts [56], which may have been a major source of fibroblasts in cardiac fibrosis in the case of transplant rejection in human patients [57]. Other evidence supporting a diverse origin of fibroblasts comes from green fluorescent protein (GFP)-labeled cells used in bone marrow transplants and experiments performed on aging mice [58]. The endothelial to mesenchymal transition may also contribute to the active pool of fibroblasts. Fibroblasts of the endothelial origin have been identified in mice carrying GFP tagged fibroblast-specific protein 1(FSP1), where lacZ marked the endothelial cells [59]. Identification and determination of fibroblast markers is a major limiting factor in the studies focused on determining the cell-of-origin for activated fibroblasts, and this compromises the systematic investigation of the relative contribution of fibroblasts from the endothelial, mesenchymal, and hematopoietic origin. The progenitor cell origin and the extent of contribution from multiple cell lineages may be pathophysiology-dependent and vary according to the degree of inflammation and the progression of the disease. Nonetheless, although the heterogeneous nature of the adventitia and aortic valve leaflets hinders our ability to distinguish between different fibroblasts originating from various progenitor cells, recent lineage studies using cell-type-specific markers, Cre-driven recombination, and cell-type-specific reported technology has advanced our understanding of the particular contribution of progenitor cells. In this section, we will discuss in detail the different cell populations that differentiate into the myofibroblast phenotype.

### 5.1. Macrophages and Monocytes

Macrophages and monocytes are highly heterogeneous cells involved in the initiation and resolution of fibrosis through a wide range of anti- and pro-fibrotic functions. Various inflammatory signals in the microenvironment of atherosclerotic plaques and fibrotic valve tissue may induce different functional and phenotypic changes in macrophages [60,61,62], e.g., IL-4- and IL-13-stimulated macrophages become M2 macrophages and are involved in fibrosis and tissue remodeling [63,64]. Furthermore, besides being under the control of cytokines and growth factors with fibrotic function, macrophages may indeed differentiate into myofibroblasts. The macrophage to myofibroblast transition (MMT) is a known phenomenon in renal fibrosis [65,66]; however, as for atherosclerosis or AVS, this sort of transition is not yet identified. Although fibroblasts of a hematopoietic origin have been identified to infiltrate injured hearts in the studies performed in chimeric mice [58,67], the specific cell-of-origin for these cells was not clear and further investigation is required to establish their monocytic origin. Macrophages are also the effector cells involved in remodeling and fibrosis because of their ability to secrete MMP-1, -7, -8, -9, and -12, as well as tissue inhibitors of MMPs (TIMPs) [61]. The proper balance of MMPs and TIMPs is crucial for the normal deposition and degradation of the ECM, which regulates the differentiation of myofibroblasts, and macrophages assist in this process by virtue of producing MMPs and TIMPs. Macrophages also contribute to the recruitment of myofibroblast to the site of injury through the ECM remodeling. They are also a leading source of TGF-β, a major pro-fibrotic agent, which induces the expression of ECM genes and suppresses the activity of genes encoding MMPs, which are capable of degrading ECM [68,69]. Therefore, macrophages promote the myofibroblast phenotype through TGF-β [70,71].

### 5.2. Endothelial to Mesenchymal Transition

EndMT was first described as a critical event in the formation of the endocardial cushion, the primordia of the valves, and septa of the adult heart [72]. Mature ECs can acquire myofibroblast-like properties through the same EndMT, during which endothelial markers are lost, and mesenchymal markers are acquired [73]. EndMT is accompanied by phenotypic and polarity changes in transformed cells, where the cells acquire a migratory phenotype and the capability to produce ECM [74]. EndMT has been described as an active contributor in the fibrosis of the heart [59], aortic valve stenosis [75,76], kidney [77], dermis [78], and atherosclerosis [9,79]. EndMT is driven by TGF-β signaling, oxidative stress, and hypoxia, which are all hallmarks of atherosclerosis. It is a multi-step process where the final mesenchymal cells may also sometimes retain the endothelial marker, which has helped in tracing the endothelial origin of these cells. Recent observations in the animal models of atherosclerosis have reported the existence of a repertoire of cells that express both mesenchymal markers (α-SMA, Snail, Vimentin, and FSP1 (S100A4)), as well as endothelial markers (VE-cadherin, PECAM-1, and Endocan), indicating a considerable endothelial origin of the neointimal cells, which may differentiate into myofibroblasts. Furthermore, lineage tracing experiments performed in animal models of atherosclerosis suggest that up to 30% of the aortic endothelial cells undergo EndMT, of which, about 50% had lost their endothelial markers, indicating a complete transformation [9,79]. Vasa vasorum, which supplies nutrients and oxygen to the large vessels, harbors endothelial progenitor cells that may serve as a reservoir of ECs for differentiation into myofibroblasts [80]. Multiple signaling/factors pathways are required in a concerted manner for the complex process of EndMT, including oxidative stress, TGF-β signaling, and β-catenin/Wnt. The canonical TGF-β/Smad signaling explained in the previous section is considered to be one of the key driving forces for EndMT [81,82]. EndMT could also be induced by the impact of blood-pressure-derived forces on the endothelial cells, these forces cause slight deformations of the artery walls, and the resulting mechanical stress induces the expression of SMC markers in ECs [83]. The extent of stress through these forces determines the course of EndMT. Different signaling pathways are activated based on the magnitude of the pathological strain. TGF-β is activated under relatively low stress, whereas β-catenin/Wnt signaling is activated by a higher magnitude strain. EndMT has also been observed in AVS, where it is driven by activation of endothelial cells by TNF-α and IL-6 through an Akt/NF-κβ-dependent pathway, as well as via TGF-β signaling [84].

### 5.3. Fibrocytes as the Source of Fibroblasts in AVS and Atherosclerosis

Fibrocytes are mesenchymal cells derived from CD14+ monocytes and have a unique phenotype. They express hematopoietic markers and produce ECM. Thus, they have features of both fibroblasts and macrophages [85]. The number of fibrocytes increases in pathologies where the persistence of myofibroblast activation and macrophage-induced inflammation is present [86]. Fibrocytes are responsive to TGF-β and differentiate into myofibroblasts upon stimulation with TGF-β or endothelin-1. In vitro, fibrocytes differentiate into the α-SMA-positive myofibroblast phenotype upon treatment with pro-fibrotic cytokines and growth factors and contribute to fibrosis in a range of pathologies including fibrosis of the kidney, lungs, and heart [87]. Pro-collagen I and CD34-positive fibrocytes have been identified in the fibrous cap of the human atherosclerotic lesions, where they contribute to plaque stability [88]; a similar observation was recapitulated in an animal model of high cholesterol diet-induced atherosclerosis [89]. In AVS, fibrocytes have been identified, but their contribution to the myofibroblast phenotype has not yet been explored [90].

## 6. Clinical Implications of Fibrosis

Development of fibrosis from a physiological to a pathological stage is a lengthy and complicated process, which is asymptomatic for a long time. Therefore, interventions at an early stage are not possible until the presentation of clinical symptoms. Recently developed methods of tissue fingerprints of fibrosis, biomarkers, and advanced imaging techniques that are used to assess and monitor the fibrosis have helped in identifying the association between fibrosis, clinical symptoms, and prognosis of the disease in multiple fibrotic pathologies, including that of atherosclerosis, heart, lungs, and liver [91]. Although further advancement of these techniques is essential to fully validate and correlate various aspects of fibrosis to the clinical evolution of the disease, several promising studies have identified ECM regulators as indicators of clinical phenotype severity. For example, in peripheral artery disease, TGF-β doubling indicated a 40% higher incidence rates of the disease and procollagen type III N-terminal propeptide (PIIINP) was associated with carotid media-intima thickness, supporting the link between ECM turnover and atherosclerosis [92].

Similarly, in subjects with nonalcoholic fatty liver disease, advanced fibrosis was associated with high carotid media-intima thickness [93]. A fibrous cap structure of carotid artery plaques is associated with a worse prognosis, and recent clinical trials have validated the significance of plaque composition as a marker for the clinical management of atherosclerosis using various imaging methods [94,95]. In the case of AVS, myocardial fibrosis is indicative of clinical outcomes and survival post valve replacement [96], and combined use of cardiac magnetic resonance and echocardiography has been proposed to assess myocardial fibrosis and left-ventricular dysfunction as a measure to determine the timing of aortic valve replacement [97].

## 7. Conclusions

Aortic valve sclerosis progresses from non-stenotic to severe stenosis through fibrosis and calcification, which increases the valve thickness from a fraction of a centimeter to over a centimeter in calcified stages. Although there is substantial literature describing the mechanism of calcification and osteogenic transformation, fibrosis alone is not that well examined. Perturbations in the mechanisms of ECM synthesis, organization, and degradation co-occur with the activation of osteogenic signaling. As extensive calcification represents advanced stage sclerosis with an attenuated valve function, it is therefore of higher importance to investigate and intervene at the early fibrotic stages. In fact, a reversal of fibrosis may be possible, which has been documented in animal models of cardiomyopathy [98,99] where the AT1 antagonist losartan reversed fibrosis and suppressed TGF-β1 expression. Admittedly, it is difficult to transpose studies performed in animal models when it comes to complex and heterogeneous structures such as atherosclerotic plaques and sclerotic valves. In vitro models using patient-derived material provide promising systems, which are used extensively to study the structure and composition of stenotic valves and plaques. Development of 3D models of the disease with various other cell populations that mimic physiological conditions will help further. Although the heterogeneous nature of the fibroblasts imposes a challenge in the systemic investigation of the mechanism of fibrosis, several signaling pathways have been shown to be universally activated in multiple in vivo and in vitro models of fibrosis. TGF-β1, IL-1β, and angiotensin II expression, in particular, have been identified as key factors. These molecules program the differentiation of progenitor cells into myofibroblasts and facilitate the maintenance of fibrosis. Although the majority of attention has been given to the identification of pro-fibrotic molecules and their inhibition, less information is available about the anti-fibrotic agents, such as, IL-10 and interferon-γ [100,101], and their role in the inhibition of myofibroblast recruitment in response to injury. More proteomics-based investigations are required to gain a complete understanding of the molecules involved in fibrosis and to identify the disease signature at various stages.
